# Power−efficient ramped stimulation in a fully implantable cochlear implant

**DOI:** 10.1038/s41598-025-25077-2

**Published:** 2025-11-21

**Authors:** Mert Doğan, Ozlem Topcu, Hasan Ulusan, M. Berat Yuksel, M. Birol Uğur, Haluk Külah

**Affiliations:** 1 Department of Electrical and Electronics Engineering, METU, Ankara, 06800 Turkey; 2 METU-MEMS Center, Ankara, 06530 Turkey; 3 Department of Otorhinolaryngology, Faculty of Medicine, Gazi University, Ankara, 06500 Turkey

**Keywords:** Cochlear implant, Ramped stimulation, Energy-efficient neurostimulation, Electrode-tissue interface, Auditory brainstem response (eABR), Biological techniques, Engineering, Neuroscience

## Abstract

Cochlear implants (CIs) are among the most established neuromodulation devices, providing auditory perception through electrical stimulation of the auditory nerve. While conventional stimulation strategies rely on symmetric biphasic rectangular pulses, alternative pulse shapes may offer improvements in neural activation and energy efficiency-particularly for fully implantable CI systems where power consumption is a key limitation. In this study, we investigate the efficacy of anodic-first ramped biphasic pulse shapes compared to conventional anodic-first rectangular pulses, using a custom-designed fully implantable cochlear implant (FICI) system in an in vivo guinea pig model. Electrically evoked auditory brainstem responses (eABRs) were recorded in response to four charge-balanced waveform types: Rectangular, RampUp, RampDown, and RampLong. In this single-subject feasibility study, ramped waveforms elicited significantly larger eABR amplitudes, steeper input-output functions, and shorter latencies compared to rectangular pulses. Additionally, we characterized transmission efficiency across the electrode–tissue interface by analyzing waveform spectra and their attenuation through a frequency-dependent medium model. After correcting for these medium-specific losses, in the anodic-first biphasic configuration, RampUp and RampLong pulses demonstrated up to 19–22% improvement in power efficiency relative to rectangular pulses at subthreshold response levels. These findings highlight the potential of ramped stimulation to reduce energy consumption without compromising-and in some cases enhancing-neural activation. Such improvements are especially valuable for fully implantable devices, supporting longer battery life and more sustainable stimulation strategies in next-generation CIs.

## Introduction

Cochlear implants (CI) are among the most successful neural prostheses, aiming to restore hearing in individuals with severe-to-profound hearing loss. In CIs, auditory perception is achieved by delivering charge-balanced electrical pulses to the primary auditory neurons via intracochlear electrodes. Conventional systems typically rely on a speech processor that must be worn externally-either behind the ear or magnetically coupled over the implant. While this architecture has proven highly effective, it comes with disadvantages such as cosmetic visibility, daily handling of external components, and limitations on continuous use (e.g. during sleep or water-based activities). To overcome these limitations, fully implantable cochlear implant (FICI) systems have been proposed. By integrating the speech processor, power supply, and microphone completely under the skin, fully implantable systems offer clear advantages in terms of discretion, convenience, and round-the-clock use. The transition to a fully implantable architecture places stringent requirements on energy efficiency, since power is supplied exclusively by rechargeable implantable batteries. In such systems, reducing power consumption directly contributes to longer battery life, fewer charging cycles, and improved patient comfort.

Conventional stimulation strategies typically used cathodic-first symmetric biphasic rectangular pulses^1^, consisting of anodic and cathodic phases of equal amplitude and duration to ensure charge neutrality and safety. While this approach is effective, it requires relatively high voltage stimulator interfaces with high-power consumption, to achieve large input dynamic range, which is quite challenging for fully implanted systems where energy efficiency is critical^2^.

In addition to the high power consumption associated with rectangular pulses^3,4^, several alternative stimulation strategies-such as triphasic waveforms^5,6^, pseudomonophasic and asymmetric pulses^7^, and waveform modifications including inter-phase gaps and polarity asymmetries^8^-have been proposed to improve the performance of intracochlear stimulation. These approaches aim to enhance spatial selectivity, reduce stimulation thresholds, and minimize unintended neural activation or inhibition. Among them, ramped biphasic pulses have recently attracted attention due to their potential physiological advantages^9^ and encouraging experimental outcomes^9–12^. In the anodic-first configuration examined in this study, ramped pulses may better align with the intrinsic dynamics of spiral ganglion neurons (SGNs), which are more responsive to gradual changes in current and exhibit more synchronized firing when exposed to stimuli with increasing amplitude^9–11^. Beyond their possible improvements in neural activation, non-rectangular waveforms with smoother transitions-such as ramped pulses-have also been shown to mitigate electrode polarization at the electrode–tissue interface^13^. Moreover, the shape of the stimulation waveform can influence the charge injection capacity (CIC) and long-term electrode integrity^14,15^, suggesting that ramped pulses could contribute to extending implant longevity and safety.

Previous studies investigating waveform shape were carried out in conventional systems or simplified stimulation setups. Fully implantable systems incorporate unique hardware constraints that can directly alter both waveform fidelity and neural response. For example, custom low-power stimulator architectures often discretize ramps into limited digital steps, which may distort the intended waveform. Limited voltage headroom can exaggerate differences in electrode voltage profiles, and the use of piezoelectric front-ends introduces frequency-dependent characteristics not present in conventional microphone–processor chains. Because of these hardware- and medium-specific factors, the same neural and efficiency results observed in previous studies cannot simply be assumed. Therefore, it is necessary to re-examine ramped stimulation in the specific context of a fully implantable system.

While stimulation performance in terms of neural dynamics has been widely studied-particularly for ramped pulses-the translation of effective stimulation into lower power consumption is especially critical for fully implantable devices. In such systems, energy is supplied by rechargeable batteries, and reducing power consumption directly contributes to longer battery life and fewer charging cycles. It has been shown that neural stimulation is by far the most power-hungry task of a CI system^16^. This imbalance in power consumption becomes even more pronounced in fully implantable systems, where ultra-low-power front-end electronics have been developed to minimize signal processing energy requirements. For instance, in the FICI system described by Ulusan et al.^4^, the analog signal chain consumes as little as a few dozen microwatts, making stimulation the dominant energy consumer. Therefore, optimizing the stimulation waveform is not only a matter of neural efficacy but a critical design consideration for extending battery life and improving the practicality of next-generation CIs, specifically by reducing recharging frequency.

In this study, we investigate whether anodic-first ramped biphasic stimulation pulses can reduce power consumption while maintaining effective auditory nerve activation, compared to conventional rectangular pulses. We hypothesize that ramped waveforms can achieve equivalent neural activation with reduced stimulation current and power compared to anodic-first conventional rectangular pulses, making them well-suited for energy-constrained, fully implantable systems. Using a FICI system with a custom-designed low-power stimulator, we evaluated multiple pulse shapes under charge-matched conditions in an in vivo model with electrically evoked auditory brainstem responses (eABRs). To our knowledge, this is the first experimental study to assess ramped pulse waveforms within the context of a FICI system, highlighting their potential to improve energy efficiency in next-generation neural prosthetics.

## Methods

This section describes the stimulation hardware, waveform design, and in vivo measurement protocol used to evaluate the efficiency of ramped and rectangular biphasic pulses in a FICI hardware system. Unlike previous in vivo studies evaluating waveform shape, this study was performed using a fully implantable system with closed-loop acoustic input and intracochlear delivery, allowing us to assess the power-performance characteristics of waveform design in a realistic, hardware-constrained context.

### FICI, stimulation IC design and operation

The custom stimulator integrated circuit (IC) used in this study was previously developed as part of a FICI system^2^. The FICI system includes a piezoelectric (PZT) acoustic transducer that senses ambient sound through the vibration of the ossicles^17^. As such, the system mimics the natural sound-processing mechanism through the multi-channel transducer. This structure filters incoming sounds into eight frequency bands via resonant piezoelectric beams, each tuned to a specific range within the auditory spectrum. During autonomous operation, the analog front-end (AFE) processes signals from the transducer and supplies sound level information to a programmable stimulation engine. Based on this input, the stimulator generates charge-balanced biphasic current pulses with various waveform shapes, including symmetric rectangular and ramped pulses.

**Fig. 1:**
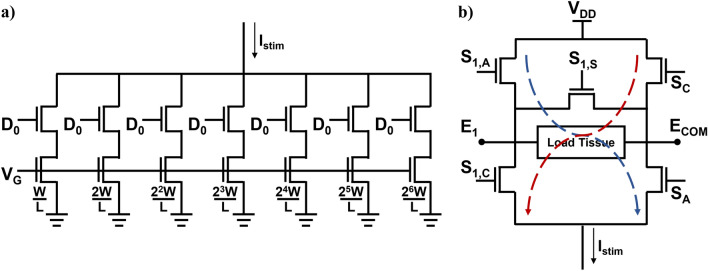
(**a**) Seven Bit DAC transistor level schematics. Upper transistors behave as switches to adjust stimulation current amount, according to bottom transistor widths. (**b**) Stimulation switch matrix. Letter coded switches operate simultaneously, choosing current paths.

Stimulation currents are produced using a 7-bit digital-to-analog converter (DAC) with a current source (Fig. 1a). The DAC-controlled gate voltage regulates the output current amplitude, enabling fine control over both threshold and maximum comfort levels. This topology supports wide-range programmability while maintaining low power consumption compared to previous-generation current generators^18,19^. The switch matrix in IC (Fig. 1b) converts the generated current into biphasic pulses and routes them to the appropriate electrode channels. Anodic pulses are delivered by sourcing current from a selected channel electrode to a common electrode (ECOM), while cathodic pulses are delivered by sinking current from the common electrode back to the same channel. Between stimulations, a shorting switch passively discharges residual charge by connecting electrodes to ECOM, providing passive charge balancing and minimizing long-term electrode polarization.

Stimulation is synchronized using a continuous interleaved sampling (CIS) strategy, enabling sequential, interleaved activation of electrodes at a fixed rate-consistent with clinically accepted CI stimulation paradigms.

The delivered current waveforms were verified by measuring electrode voltages across the electrodes using an oscilloscope, enabling monitoring of the actual stimulation currents and confirmation of the intended pulse shapes. The current source operated with a 12 V supply and provided a compliance voltage of up to 10.8 V across the electrode–tissue interface, which was sufficient for all tested stimulation conditions.

### Stimulation waveforms and efficiency calculation

Four anodic-first biphasic stimulation waveforms were evaluated: a conventional rectangular pulse and three ramped variants (RampUp, RampDown, and RampLong). This choice was made to remain consistent with prior work on ramped pulses^9,11^ and to align with the configuration supported by the present FICI hardware. The waveforms were charge-balanced, with 50 $\upmu$s phase durations (100 $\upmu$s total), and tested under charge-matched conditions to isolate the effect of waveform shape on stimulation efficiency. The four waveform types, evaluated under identical duration, were defined as follows:

Rectangular – A symmetric biphasic waveform with constant amplitude in each phase.

RampUp – A linearly increasing current during each phase, from 0 $\mu$A to the peak value.

RampDown – A linearly decreasing current during each phase, from peak to 0 $\mu$A.

RampLong – A slope-asymmetric waveform with equal-duration phases (50 $\mu$s each). The anodic phase ramps down from the peak current to 0 $\mu$A, while the cathodic phase ramps up from 0 to −peak $\mu$A. Despite this directional asymmetry, total charge was balanced between phases.

**Fig. 2:**
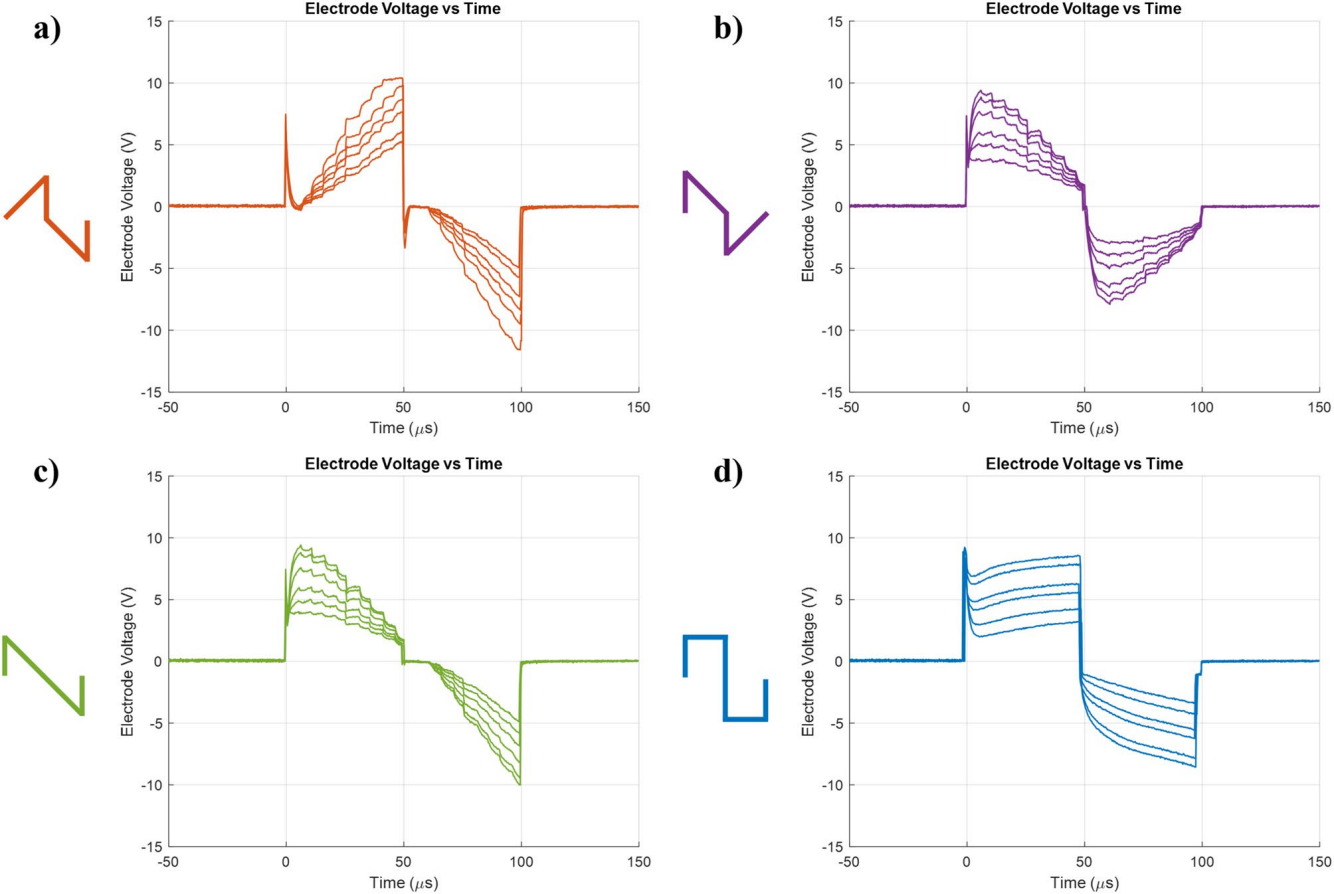
Experimental stimulation site voltage waveforms on the electrode for 4 pulse shapes. (Initial spike-like behaviour is observed to be a switching artifact due to capacitive charging.) (**a**) RampUp (**b**) RampDown (**c**) RampLong (**d**) Rectangular. Each trace corresponds to different charge level, from 50 to 100 dB.

Waveform generation was digitally controlled via a microcontroller, which set DAC values corresponding to stimulation intensities derived from representative sound level inputs. Each 50 $\mu$s phase was discretized into 10 equal segments of 5 $\mu$s. No additional filtering or smoothing was applied; thus, any observed waveform deformation was due to the capacitive characteristics of the electrode–tissue interface as observed in Fig. 2.

To ensure fair comparisons, total charge per phase was matched across waveform types. For instance, a ramped waveform with a 50 $\mu$A peak current delivered the same total charge as a rectangular waveform with 25 $\mu$A constant amplitude. Pulses were evaluated for a custom dB scale, charge injection levels from 50 to 100 dB where 50 dB increase corresponds to doubled current.

Stimulation efficiency was defined as the relative reduction in stimulation power required to evoke a comparable eABR amplitude, expressed as a ratio with respect to the conventional rectangular pulse. For each waveform, the effective (RMS) stimulation current was calculated over the pulse duration, and power per pulse was estimated as $P = V \times I_{rms} \times t$, where *V* is the compliance voltage (12 V) and *t* is the pulse duration. Efficiency was then quantified as ($P_{rectangular}-P_{waveform}) / P_{rectangular} \times 100\%$, such that more positive values indicate reduced power requirements compared to the rectangular baseline.

### Electrode interface modelling

To assess the electrical transmission efficiency of each stimulation waveform independent of physiological response, we modeled the relationship between stimulation current and electrode voltage using a measured electrode–medium impedance. The electrode–tissue interface was modeled as a series RC circuit, a common approximation in cochlear implant literature^15^, representing the resistive and capacitive elements of the surrounding medium.

In the experiment, the impedance between a stimulation channel and ECOM was measured using a commercially available LCR (inductance–capacitance–resistance) meter (Agilent E4980A, USA) which provided the values of resistance R and capacitance C, representing the series path from the channel electrode to ECOM through the biological medium. Under this model, the voltage across the electrode interface can be expressed as

1 $$\begin{aligned} V_{\text {EL}}(t) - V_{\text {ECOM}}(t) = I_{\text {stim}}(t) \cdot \left( R + \frac{1}{C} \int _0^t I_{\text {stim}}(\tau ) \, d\tau \right). \end{aligned}$$

Given that the same current source drives all waveform shapes, waveform-specific differences in $I_{stim}$ (t) result in different instantaneous voltage profiles and delivered energy, even under equal total charge conditions.The four stimulation waveforms tested in vivo can be mathematically defined as follows (with total phase durations of 50 $\mu$s):

- **Rectangular**
  2 $$\begin{aligned} I(t) = {\left\{ \begin{array}{ll} +I_{\text {max}}, & \text {for } 0< t< 50\,\mu \text {s}, \\ -I_{\text {max}}, & \text {for } 50< t < 100\,\mu \text {s}, \end{array}\right. } \end{aligned}$$
- **Rampup**
  3 $$\begin{aligned} I(t) = {\left\{ \begin{array}{ll} \displaystyle 2I_{\text {max}} \cdot \frac{t}{50}, & \text {for } 0< t< 50\,\mu \text {s}, \\ \displaystyle -2I_{\text {max}} \cdot \frac{t - 50}{50}, & \text {for } 50< t < 100\,\mu \text {s}, \end{array}\right. } \end{aligned}$$
- **Rampdown**
  4 $$\begin{aligned} I(t) = {\left\{ \begin{array}{ll} \displaystyle 2I_{\text {max}} \cdot \frac{50 - t}{50}, & \text {for } 0< t< 50\,\mu \text {s}, \\ \displaystyle -2I_{\text {max}} \cdot \frac{100 - t}{50}, & \text {for } 50< t < 100\,\mu \text {s}, \end{array}\right. } \end{aligned}$$
- **Ramplong**
  5 $$\begin{aligned} I(t) = {\left\{ \begin{array}{ll} \displaystyle 2I_{\text {max}} \cdot \frac{50 - t}{50}, & \text {for } 0< t< 50\,\mu \text {s}, \\ \displaystyle -2I_{\text {max}} \cdot \frac{t - 50}{50}, & \text {for } 50< t < 100\,\mu \text {s}. \end{array}\right. } \end{aligned}$$

**Fig. 3:**
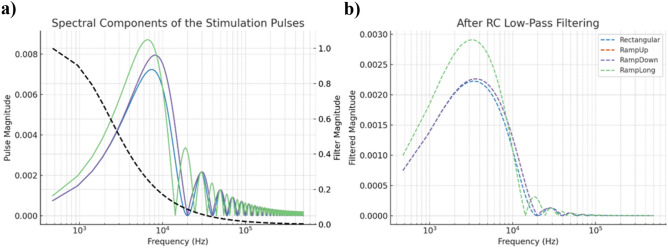
(**a**) For the tested stimulation waveforms, frequency components are calculated. Dashed line illustrates the RC filter response. At the first glance it is seen that Ramped waveform consist more low frequencies components compared to rectangular pulse. (**b**) Magnitude response of the stimulation waveforms after filtering. RampLong is suspect to less attenuation compared other waveforms.

For the charge-matched cases, the attenuations of the stimulation pulses are calculated in frequency domain to compare the ratios of actual delivered charges to the electrode-medium interface, which can affect the stimulation efficiency besides the physiological possible advantages of ramped pulses. Without the low-pass effect of the medium, for $\hbox {I}_{{max}}$ = 100 $\mu$ A, the frequency domain magnitudes of the stimulation pulses are illustrated in Fig. 3a.

### In vivo experimental setup

To evaluate the stimulation efficiency of different biphasic waveform shapes using the FICI system, in vivo experiments were conducted in one adult male Duncan-Hartley guinea pig. All procedures were approved by the KOBAY DHL A.S. Local Ethics Committee (protocol number: 399/2019) and conformed to the World Medical Association Declaration of Helsinki guidelines.

During all surgical and recording procedures, the animal was anesthetized with ketamine (40 mg/kg) and xylazine (5 mg/kg), administered intraperitoneally. Anesthesia depth was monitored throughout the experiment, and supplemental doses were given as needed. The animal was housed under controlled temperature conditions (21${\pm }1^{\circ }$C) with a 12 h reverse light–dark cycle and had free access to food and water. For hydration during the experiments, 10 mL/kg intraperitoneal saline was administered.

**Fig. 4:**
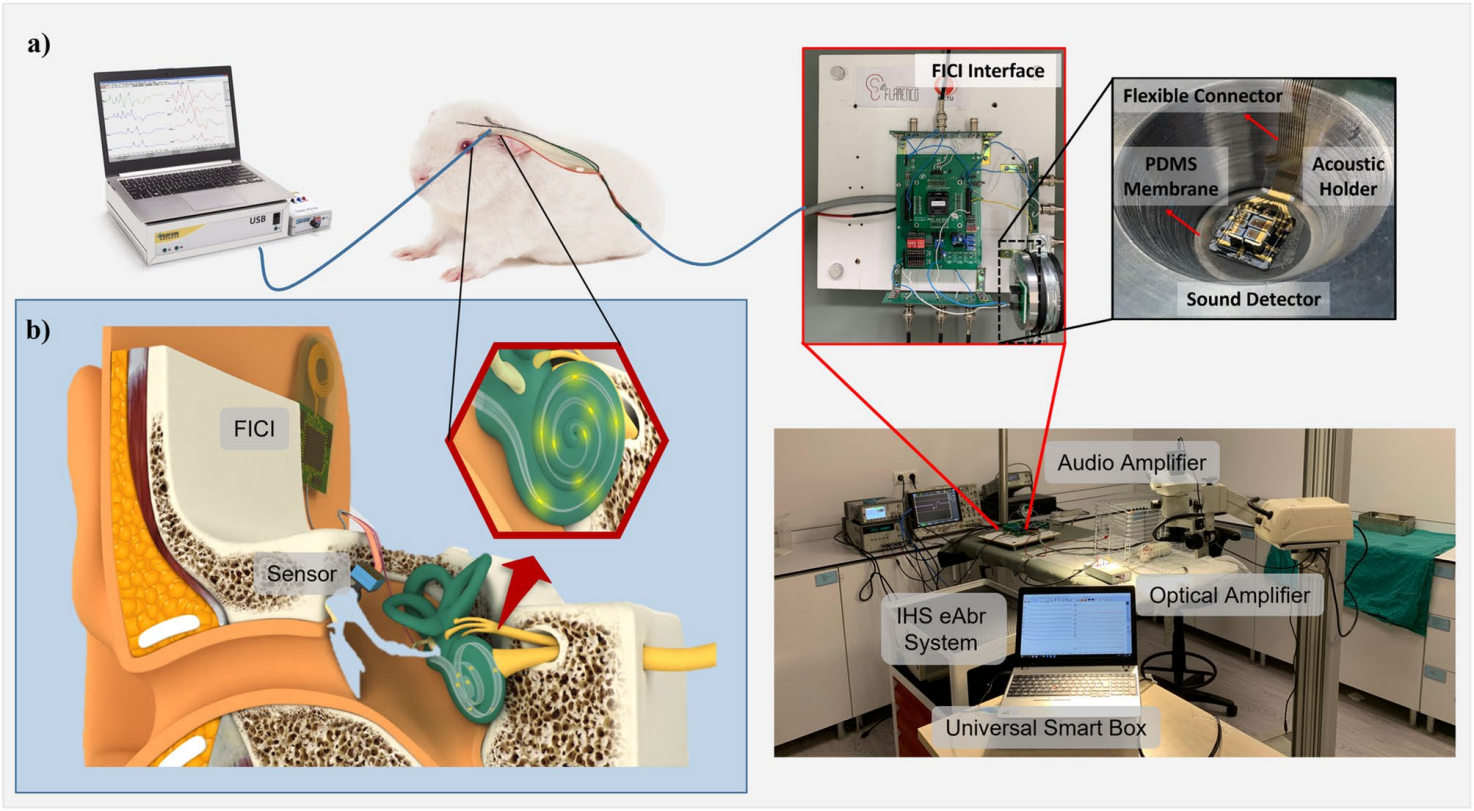
(**a**) The FICI system and schematic diagram of the in vivo test procedure. Intracochlear electrodes were surgically placed to the cochlea of the animal model. Piezoelectric transducer was placed on an artificial membrane mimicking the ear canal.Ramped and rectangular biphasic current pulse are generated by the FICI system to stimulate the primary auditory neurons of the guinea pig. The current pulses are provided through an intracochlear electrode array placed into the scala tympani of the cochlea, inserted through the round window. The stimulation response was observed eABR measurements, through the optical amplifier and the Universal Smart Box of Intelligent Hearing Systems (HIS) (**b**) The FICI hardware system and sensor are shown in their intended implantation locations on a human head cross-section. The FICI housing, which contains the hardware, battery, and wireless power transfer module, is positioned on the mastoid bone, while the sensor is placed on the middle ear ossicular chain.

The experimental setup is shown in Fig. 4a. A low-profile piezoelectric (PZT; lead zirconate titanate) sound detector was mounted on a thin PDMS (polydimethylsiloxane) membrane, a biocompatible elastomer widely used in biomedical devices. In this setup, the PDMS membrane was placed within a 10 mm diameter, 2 cm deep acoustic holder designed to mimic the ear canal. Acoustic stimulation was delivered via an Etymotic Research ER-2 insert earphone positioned at the opening of the sensor holder. Vibrations were converted into electrical signals by the PZT sensor and processed by the signal conditioning circuit of the FICI system which then generated charge-balanced biphasic current pulses for stimulation. Figure 4b illustrates the intended anatomical placement of the FICI in a human model for orientation only; it was not part of the animal experiment.

A commercially available intracochlear electrode array (MED-EL GmbH, Innsbruck, Austria) was inserted through the round window into the scala tympani. The electrode array included two intracochlear stimulation electrodes and a reference electrode placed extra-tympanically on the bony wall of the bulla. The stimulation electrodes were connected to two independent output channels of the stimulator IC and used to deliver different waveform types under charge-matched conditions.

To minimize electrophonic artifacts in auditory brainstem responses, ototoxic deafening was performed on the experimental animal by intramuscular injection of kanamycin (600 mg/kg) followed by intraperitoneal administration of furosemide (75 mg/kg) after one hour. Successful deafening was confirmed by the absence of click- and tone burst-evoked ABR responses prior to electrical stimulation.

While the FICI system was designed as a fully implantable architecture, in this feasibility study the electronic module was externalized to allow for monitoring and adjustments, while the intracochlear electrode array was surgically implanted. Thus, the stimulation and recording were carried out under realistic FICI conditions, but with the electronics externalized for experimental control.

### eABR recording and data analysis

eABR were recorded to evaluate the neural activation elicited by different biphasic stimulation waveforms. Recordings were conducted in a sound-attenuated and electromagnetically shielded booth. The non-inverting (positive) electrode was placed at the vertex, the inverting (negative) electrode over the ipsilateral superior post-auricular area, and the ground electrode in the ipsilateral hindlimb musculature. All electrode impedances were measured prior to recording and verified to be below 1 k$\Omega$, ensuring stable recordings throughout the acute experiment. To minimize electromagnetic interference, leads were twisted.

eABRs were acquired using an Opti-Amp bio-amplifier and the Universal Smart Box (Intelligent Hearing Systems, USA). Signals were digitized at 32 kHz and bandpass filtered from 30 to 3000 Hz. Each response was averaged over 512 sweeps at a stimulation rate of 21.1 Hz. Utilization of a slow stimulation rate enables separation of the temporal residual effects of stimulation pulses^20^. Stimuli were sampled at 80 kHz and recordings at 40 kHz. Wave II was used to define the eABR threshold, while waves II–IV were used to evaluate waveform-specific effects on amplitude, slope, and latency. Wave II amplitude was determined as the peak-to-trough voltage difference, measured between the most positive deflection of wave II and the subsequent negative trough, between 1.5 and 2.5 ms.

Statistical analyses were performed using SPSS$\circledR$ (version 13; SPSS Inc., Chicago, IL). Normality of data distribution was assessed using the Kolmogorov–Smirnov test. Given the non-normal distribution, a nonparametric Friedman test was applied, followed by Bonferroni-adjusted Wilcoxon signed-rank tests for post hoc comparisons. A p-value of $\le$ 0.05 was considered statistically significant.

Given that this was a single-subject feasibility study aimed at characterizing waveform efficiency within a functional FICI system, statistical comparisons were interpreted cautiously and intended to highlight directional trends.

## Results

**Fig. 5:**
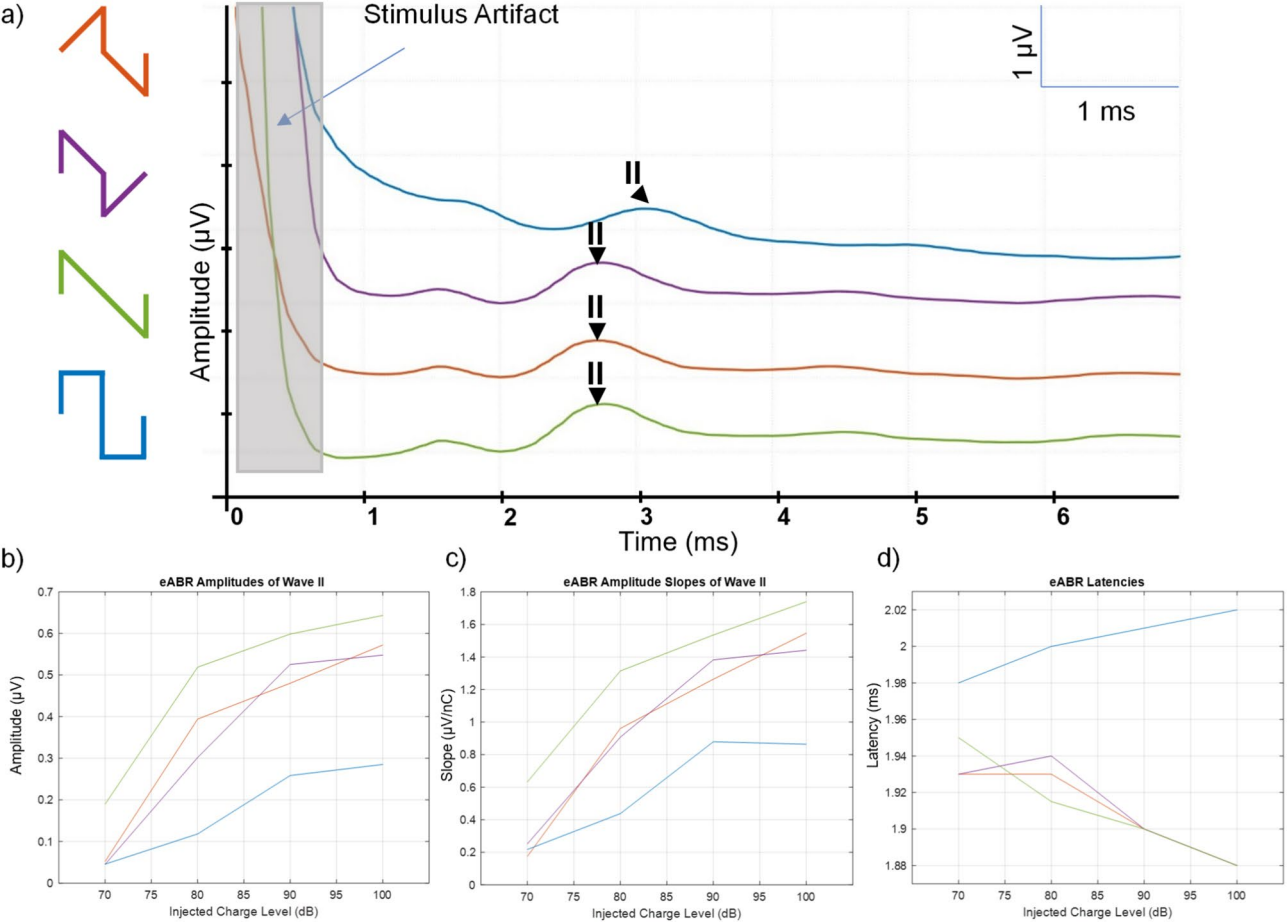
(**a**) Ramped stimulation waveform can elicit eABRs. Example responses Rectangular (blue), RampDown (purple), RampUp (orange) and RampLong (green), respectively from intracochlear electrode. Ramped pulse shapes provide a higher amplitude and steeper slope input/output function of eABR wave II compared to rectangular shapes in terms of amount of injected charge. (**b**) eABR input–output growth functions (wave II amplitude as a function of injected charge) for each waveform. Ramped pulses show larger amplitudes and clearer separation between waveform types compared to rectangular. (**c**) Slopes of the input–output functions ($\upmu$V/nC), highlighting the faster recruitment of neural responses with ramped pulses. (**d**) eABR latencies obtained at each current level for all stimulation waveforms in guinea pigs.

eABR responses were successfully recorded in response to all four stimulation waveforms. Representative recordings are shown in Fig. 5a. All ramped waveforms elicited clear wave II responses, with visibly larger amplitudes compared to the conventional rectangular pulse.

All ramped waveforms exhibited steeper amplitude growth compared to the rectangular pulse, with RampLong showing the strongest overall increase. These differences are further quantified by the slope analysis, confirming that ramped pulses recruit neural responses more efficiently as charge level increases.

### eABR amplitude

Across increasing current levels, all ramped waveforms (RampUp, RampDown, RampLong) produced significantly higher wave II peak-to-peak amplitudes than the rectangular waveform (Fig. 5b). A Friedman test indicated a significant main effect of waveform type on amplitude ($X^2$ = 20.250, *p* < 0.0001). Post hoc Wilcoxon signed-rank tests (standardized statistic value of Z) showed significant differences between Rectangular and RampUp (Z = –2.521, *p* = 0.012), RampDown (Z = –2.240, *p* = 0.025), and RampLong (Z = –2.521, *p* = 0.012). RampLong also differed significantly from RampUp (Z = –2.521, *p* = 0.012) and RampDown (Z = –2.521, *p* = 0.012), while RampUp and RampDown did not differ significantly from each other (Z = –0.700, *p* = 0.484).

### Input–output slope

Ramped waveforms produced steeper input–output (I/O) growth functions compared to rectangular pulses (Fig. 5c), indicating faster neural recruitment, consistent with the prior reports^9,11^. Post hoc analysis showed significant slope differences between Rectangular and RampUp (Z = –2.240, *p* = 0.025), RampDown (Z = –2.380, *p* = 0.017), and RampLong (Z = –2.521, *p* = 0.012). RampLong also differed significantly from both RampUp (Z = –2.521, *p* = 0.012) and RampDown (Z = –2.521, *p* = 0.012), while RampUp and RampDown were not significantly different (Z = –0.280, *p* = 0.779).

### Latency

All ramped pulses showed significantly shorter wave II latencies compared to rectangular stimulation (Fig. 5d), suggesting faster or more synchronized neural activation^9,11^. Latency differences were statistically significant between Rectangular and RampUp (Z = –2.530, *p* = 0.011), RampDown (Z = –2.536, *p* = 0.011), and RampLong (Z = –2.530, *p* = 0.011). No significant differences were observed among the ramped waveforms (all *p* > 0.05).

### Efficiency analysis

To evaluate stimulation efficiency, we compared the stimulus level required to reach equal neural responses (0.20 $\mu$V and 0.25 $\mu$V) across waveforms. The FICI system implements a custom current-scaling relationship, where every 50 dB increase in input sound level corresponds to a 2 time increase in stimulation current. This allows conversion of interpolated dB values to relative stimulation currents using the equation:

6 $$\begin{aligned} I_{\text {rel}} = 2^{\frac{x - x_{\text {ref}}}{50}}, \end{aligned}$$

where *x* is the interpolated dB for the waveform, and $x_{\text {ref}}$ is either the rectangular value (for relative current) or the stimulation threshold (for true above-threshold comparisons). The threshold for all waveforms was experimentally determined to be 45 dB. Thresholds were determined from the interpolated eABR input–output functions shown in Fig. 5b, with wave II amplitude used as the defining criterion.

Table 1 summarizes the interpolated dB levels for each waveform, the corresponding dB above threshold, and the relative stimulation current required to achieve the same response. Efficiency gain was calculated as the percentage reduction in required current compared to the rectangular pulse.

These results clearly demonstrate that anodic-first biphasic ramped stimulation waveforms - particularly RampLong - require significantly less current above threshold to evoke equivalent neural responses. At 0.20 $\mu$V, RampLong achieved a 30.1$\%$ reduction in stimulation current compared to rectangular pulses; even at the higher 0.25 $\mu$V response level, current savings remained above 28$\%$.

During the experiments, series resistance R is measured as 8.45 kOhm and series capacitance is measured as 11.25 nF. According to these values, frequency domain magnitudes for the stimulation waveforms are calculated and plotted (3b). It is seen that RampLong pulses attenuates the least, followed by RampUp and RampDown pulses. As summarized in Table 2., RampLong pulses can inject 22$\%$ more charge to the medium than Rectangular pulses, while RampUp and RampDown pulses can supply 12$\%$ more charge compare to Rectangular pulses. Current corrections are given by the ratio of the total current amount in frequency domain after filtering to prefiltered total current amount in frequency domain. For power correction, squared currents are utilized. Rectangular pulse current and power levels are selected as the baseline.

**Table 1:** Comparison of waveform responses at different fixed voltage levels and thresholds.

Response	Waveform	Interpolated dB	dB above threshold	Relative current	Efficiency gain (%)
**0.20** $\mu$**V**	RampLong	71.0	26.0	1.78	**30.1%**
	RampUp	73.6	28.6	1.90	**25.4%**
	RampDown	76.5	31.5	2.05	**19.6%**
	Rectangular	86.0	41.0	2.55	—
**0.25** $\mu$**V**	RampLong	72.1	27.1	1.83	**28.3%**
	RampUp	76.5	31.5	2.05	**19.6%**
	RampDown	77.6	32.6	2.11	**17.3%**
	Rectangular	88.2	43.2	2.73	—

**Table 2:** Attenuation-corrected power efficiency gains and relative attenuation factors across different waveforms.

Waveform	Current attenuation factor	Power attenuation factor	Efficiency gain (0.20 $\mu$V)	Efficiency gain (0.25 $\mu$V)
	(Relative to rectangular)	(Relative to rectangular)	(%)	(%)
RampLong	0.82	0.65	**19.6**	**18.4**
RampUp	0.88	0.88	**22.4**	**17.2**
RampDown	0.88	0.88	**17.2**	**15.2**
Rectangular	1.00	1.00	—	—

## Discussion

This feasibility study demonstrates that anodic-first ramped biphasic stimulation waveforms-particularly RampLong-consistently evoke stronger auditory nerve responses compared to conventional rectangular pulses under charge-matched conditions. This is evidenced by significantly higher eABR amplitudes, steeper input-output growth functions, and shorter wave latencies observed in response to ramped pulses.

A likely explanation lies in the underlying biophysics of SGNs. SGNs exhibit sensitivity to the rate of change in depolarizing currents, with low-threshold potassium channels playing a central role in shaping firing precision and adaptation^21^. Waveforms with smoother current transitions, such as ramped pulses, appear to engage these mechanisms more effectively-enhancing phase-locking and reducing temporal jitter, as previously demonstrated^22^. This increased synchrony across auditory fibers likely contributes to the larger eABR amplitudes and steeper growth functions observed in this study. The shorter response latencies observed with ramped pulses further support this interpretation. Latency is a composite marker influenced by membrane time constants, ion channel activation thresholds, and recruitment patterns along the auditory nerve. Ramped pulses may provide a more efficient trajectory to threshold crossing, enabling faster and more coordinated firing, which is critical for precise temporal coding of acoustic signals^23^.

Notably, although RampUp and RampDown both use sloped current profiles, RampLong-which combines a descending anodic and an ascending cathodic phase-elicited the most robust responses. While RampDown also contains a descending anodic phase, RampLong uniquely provides both a down-ramp anodic and an up-ramp cathodic transition. This combination may facilitate stronger phase interactions, as partial depolarization from the anodic phase can be reinforced by the rising cathodic phase. Zheng et al.^24^ reported in cathodic-first configurations that recovery from hyperpolarisation block can enhance responsiveness, underscoring that phase interactions can strongly influence recruitment mechanisms. Such biphasic cooperativity may enhance spike probability especially in borderline threshold conditions, as has been shown in computational models of auditory nerve activation.

When accounting for waveform transmission efficiency through the electrode–tissue interface, the interpretation becomes more nuanced. Due to differences in spectral content, each pulse shape undergoes varying levels of attenuation as it propagates through the biological medium. After normalizing for this attenuation, RampUp and RampLong pulses exhibit comparable power efficiency gains relative to the rectangular baseline. Specifically, RampUp and RampLong both achieve attenuation-corrected power efficiency gains of approximately 19–22$\%$ at sub-threshold response levels (Table 2), with RampDown trailing slightly behind. This finding suggests that RampLong’s superior raw response is at least partially attributable to better medium transmission, rather than purely intrinsic neural dynamics. Once this factor is isolated, RampUp emerges as similarly effective-and potentially more energy efficient due to reduced power loss during transmission.

These results align with and expand upon several prior investigations into ramped stimulation waveforms. Navntoft et al.^9^ demonstrated in a guinea pig model that ramped pulse shapes produced stronger eABR responses than conventional rectangular pulses, a finding mirrored in our own amplitude and slope comparisons. In follow-up work, Navntoft et al.^10^ further reported that ramped pulses are perceived as louder by human CI users-likely a behavioral correlate of the increased neural synchrony we observed in animal recordings.

On a cellular level, Ballestero et al.^11^ showed that the firing probability of SGNs increases with a gradually ramping input, consistent with our observation that ramped pulses more effectively recruit neural populations at lower current levels. This mechanistic insight helps explain the steeper eABR input-output slopes we report, especially for RampUp and RampLong. Additionally, Partouche et al.^12^ evaluated ramped waveforms in auditory cortex responses and found improved spiking precision and cortical recruitment. While their work emphasized central processing, our findings suggest that improved phase-locking and lower temporal jitter-already evident at the auditory nerve level-may propagate along the ascending auditory pathway, contributing to the observed benefits in higher centers.

Our study also provides a novel perspective by incorporating medium-related attenuation into waveform efficiency analysis. While Yip et al.^16^ previously optimized stimulation shapes using nerve models, their work did not account for transmission losses at the electrode–tissue interface. By integrating spectral attenuation into our framework, we offer a more realistic, experimentally grounded evaluation of waveform performance in vivo.

Our findings indicate that ramped stimulation waveforms, particularly RampUp and RampLong, offer significant power efficiency gains over traditional rectangular pulses. After correcting for medium attenuation, both anodic-first RampUp and anodic-first RampLong demonstrate approximately 19–22$\%$ improvements in power efficiency at sub-threshold response levels. This enhancement translates to a proportional reduction in the energy required for neural stimulation, thereby extending the operational lifespan of the implant between charges. The neural stimulation component of the FICI system^2^ consumes 580 $\upmu$W with rectangular pulses, adopting ramped waveforms could reduce this consumption to approximately 460 $\upmu$W. This decrease not only prolongs battery life but also aligns with the thermal and safety constraints associated with the implantable battery, as lower power consumption mitigates heat generation during operation and allows for more conservative charging phases, crucial for FICIs. Importantly, improved energy efficiency translates into less frequent charging, which not only enhances user comfort and convenience but also contributes to longer battery longevity. Since implantable batteries have a limited number of charge-discharge cycles before requiring surgical replacement, reducing the charging frequency directly supports device sustainability and long-term patient well-being.

This study presents an early proof-of-concept, demonstrating that anodic-first ramped stimulation pulses can improve neural efficiency compared to traditional rectangular pulses in a FICI system. However, these results were obtained from a single animal, so further experiments across multiple subjects are needed to confirm how consistent and reliable the improvements are.

There are also some technical considerations when it comes to implementing ramped pulses in hardware. Generating smooth waveforms like ramped shapes usually requires more control points in each stimulation phase, which in turn demands a higher-resolution DAC and faster update rates. While this allows for better control of the waveform, it also means more power consumption, which is an important limitation for low-power, fully implantable devices. So, designers must find a balance between precision and efficiency. In our results, we also observed that RampUp pulses can briefly lead to higher electrode voltages than rectangular pulses, even when the total charge is the same. This could require higher supply voltages or more headroom in the stimulator design. It’s worth investigating how this might affect electrode safety and reversibility, especially in long-term use.

Other waveform shapes, like Gaussian or sinusoidal pulses, have been explored in earlier studies as well. However, their performance often depends on how finely the waveform can be broken into digital steps-and this again leads to higher demands on hardware. So even though they might have potential, simpler shapes like ramps may be more practical, especially in systems where power and chip area are limited. Looking forward, it may be useful to move beyond fixed waveform shapes altogether. Future systems could explore adaptive stimulation, where the pulse shape is adjusted depending on electrode impedance or neural feedback. Adaptive stimulation could, in principle, involve tailoring pulse shape at activation based on electrode impedance and early neural responsiveness. While impedance stabilizes long term, adaptation at fitting could ensure optimal alignment between delivered waveform and tissue characteristics. This way, the stimulation could be optimized for each user-or even each electrode-based on how well the signal is getting through and how the nerve responds. It’s a promising direction for improving both energy efficiency and patient outcomes in next-generation CIs.

It should be noted that these results were obtained in a single animal. This limitation was deliberate, as the primary goal was to establish feasibility and characterize stimulation efficiency in the context of a FICI. Multi-subject studies will be required to validate reproducibility and generalize the findings

## Conclusion

In summary, this single-subject feasibility study demonstrates that anodic-first ramped biphasic stimulation waveforms -particularly RampUp and RampLong- can elicit stronger neural responses with lower power requirements compared to conventional rectangular pulses when tested in the context of a fully implantable cochlear implant system. Importantly, by incorporating electrode–tissue interface attenuation into the analysis, we provide the first in vivo evidence that ramped pulses offer up to 22% power efficiency gains relative to rectangular pulses under realistic implant conditions. These findings highlight the potential of ramped pulses to reduce stimulation energy in fully implantable systems, extending battery life and supporting more sustainable stimulation strategies. Further multi-subject studies will be necessary to validate these preliminary results and explore their implications for long-term clinical application.

## Data Availability

The datasets used and/or analyzed during the current study are available from the corresponding author on reasonable request.

## References

[1] Zeng, F.-G., Rebscher, S., Harrison, W., Sun, X. & Feng, H. Cochlear implants: System design, integration, and evaluation. *IEEE Rev. Biomed. Eng.* **1**, 115–142. 10.1109/rbme.2008.2008250 (2008). 19946565 10.1109/RBME.2008.2008250 PMC2782849

[2] Ulusan, H. et al. A full-custom fully implantable cochlear implant system validated in vivo with an animal model. *Commun. Eng.*10.1038/s44172-024-00275-4 (2024). 39277675 10.1038/s44172-024-00275-4 PMC11401833

[3] Wongsarnpigoon, A. & Grill, W. M. Energy-efficient waveform shapes for neural stimulation revealed with a genetic algorithm. *J. Neural Eng.* **7**, 046009. 10.1088/1741-2560/7/4/046009 (2010). 20571186 10.1088/1741-2560/7/4/046009 PMC2925408

[4] Ulusan, H., Chamanian, S., Ilik, B., Muhtaroglu, A. & Kulah, H. Fully implantable cochlear implant interface electronics with 51.2- w front-end circuit. *IEEE Trans. Very Large Scale Integr. VLSI Syst.* **27**, 1504–1512. 10.1109/tvlsi.2019.2898873 (2019).

[5] Bonnet, R. M., Frijns, J. H., Peeters, S. & Briaire, J. J. Speech recognition with a cochlear implant using triphasic charge-balanced pulses. *Acta Otolaryngol.* **124**, 371–375. 10.1080/00016480410031084 (2004). 15224856 10.1080/00016480410031084

[6] Braun, K., Walker, K., Sörth, W., Löwenheim, H. & Tropitzsch, A. Triphasic pulses in cochlear implant patients with facial nerve stimulation. *Otol. Neurotol.* **40**, 1268–1277. 10.1097/mao.0000000000002398 (2019). 31469792 10.1097/MAO.0000000000002398

[7] de Nobel, J., Kononova, A. V., Briaire, J. J., Frijns, J. H. & Bäck, T. H. Optimizing stimulus energy for cochlear implants with a machine learning model of the auditory nerve. *Hear. Res.* **432**, 108741. 10.1016/j.heares.2023.108741 (2023). 36972636 10.1016/j.heares.2023.108741

[8] Ramekers, D. et al. Auditory-nerve responses to varied inter-phase gap and phase duration of the electric pulse stimulus as predictors for neuronal degeneration. *J. Assoc. Res. Otolaryngol.* **15**, 187–202. 10.1007/s10162-013-0440-x (2014). 24469861 10.1007/s10162-013-0440-x PMC3946144

[9] Navntoft, C. A., Marozeau, J. & Barkat, T. R. Ramped pulse shapes are more efficient for cochlear implant stimulation in an animal model. *Sci. Rep.*10.1038/s41598-020-60181-5 (2020). 32094368 10.1038/s41598-020-60181-5 PMC7039949

[10] Navntoft, C. A., Landsberger, D. M., Barkat, T. R. & Marozeau, J. The perception of ramped pulse shapes in cochlear implant users. *Trends Hear.*10.1177/23312165211061116 (2021). 34935552 10.1177/23312165211061116 PMC8724057

[11] Ballestero, J. et al. Reducing current spread by use of a novel pulse shape for electrical stimulation of the auditory nerve. *Trends Hear.*10.1177/2331216515619763 (2015). 26721928 10.1177/2331216515619763 PMC4771040

[12] Partouche, E., Adenis, V., Stahl, P., Huetz, C. & Edeline, J.-M. What is the benefit of ramped pulse shapes for activating auditory cortex neurons? an electrophysiological study in an animal model of cochlear implant. *Brain Sci.* **13**, 250. 10.3390/brainsci13020250 (2023). 36831793 10.3390/brainsci13020250 PMC9954719

[13] Sahin, M. & Tie, Y. Non-rectangular waveforms for neural stimulation with practical electrodes. *J. Neural Eng.* **4**, 227–233. 10.1088/1741-2560/4/3/008 (2007). 17873425 10.1088/1741-2560/4/3/008 PMC3759998

[14] Cogan, S. F. Neural stimulation and recording electrodes. *Annu. Rev. Biomed. Eng.* **10**, 275–309. 10.1146/annurev.bioeng.10.061807.160518 (2008). 18429704 10.1146/annurev.bioeng.10.061807.160518

[15] Merrill, D. R., Bikson, M. & Jefferys, J. G. Electrical stimulation of excitable tissue: Design of efficacious and safe protocols. *J. Neurosci. Methods* **141**, 171–198. 10.1016/j.jneumeth.2004.10.020 (2005). 15661300 10.1016/j.jneumeth.2004.10.020

[16] Yip, M., Bowers, P., Noel, V., Chandrakasan, A. & Stankovic, K. M. Energy-efficient waveform for electrical stimulation of the cochlear nerve. *Sci. Rep.*10.1038/s41598-017-13671-y (2017). 29051546 10.1038/s41598-017-13671-y PMC5648926

[17] Yuksel, M. B., Atik, A. C. & Kulah, H. Piezoelectric multi?channel bilayer transducer for sensing and filtering ossicular vibration. *Adv. Sci.*10.1002/advs.202308277 (2024). 10.1002/advs.202308277 PMC11040378 38380504

[18] Yip, M., Jin, R., Nakajima, H. H., Stankovic, K. M. & Chandrakasan, A. P. A fully-implantable cochlear implant soc with piezoelectric middle-ear sensor and arbitrary waveform neural stimulation. *IEEE J. Solid-State Circuits* **50**, 214–229. 10.1109/jssc.2014.2355822 (2015). 26251552 10.1109/JSSC.2014.2355822 PMC4523309

[19] Bhatti, P. T. & Wise, K. D. A 32-site 4-channel high-density electrode array for a cochlear prosthesis. *IEEE J. Solid-State Circuits* **41**, 2965–2973. 10.1109/jssc.2006.884862 (2006).

[20] Boulet, J., White, M. & Bruce, I. C. Temporal considerations for stimulating spiral ganglion neurons with cochlear implants. *J. Assoc. Res. Otolaryngol.* **17**, 1–17. 10.1007/s10162-015-0545-5 (2016). 26501873 10.1007/s10162-015-0545-5 PMC4722016

[21] Adamson, C. L., Reid, M. A., Mo, Z., Bowne-English, J. & Davis, R. L. Firing features and potassium channel content of murine spiral ganglion neurons vary with cochlear location. *J. Comp. Neurol.* **447**, 331–350. 10.1002/cne.10244 (2002). 11992520 10.1002/cne.10244

[22] Rutherford, M. A., Chapochnikov, N. M. & Moser, T. Spike encoding of neurotransmitter release timing by spiral ganglion neurons of the cochlea. *J. Neurosci.* **32**, 4773–4789. 10.1523/jneurosci.4511-11.2012 (2012). 22492033 10.1523/JNEUROSCI.4511-11.2012 PMC6620899

[23] Trussell, L. O. Synaptic mechanisms for coding timing in auditory neurons. *Annu. Rev. Physiol.* **61**, 477–496. 10.1146/annurev.physiol.61.1.477 (1999). 10099698 10.1146/annurev.physiol.61.1.477

[24] Zheng, L., Feng, Z., Xu, Y., Yuan, Y. & Hu, Y. An anodic phase can facilitate rather than weaken a cathodic phase to activate neurons in biphasic-pulse axonal stimulations. *Front. Neurosci.*10.3389/fnins.2022.823423 (2022). 35368280 10.3389/fnins.2022.823423 PMC8968170

